# Author Correction: Synchronous, Crosstalk-free Correlative AFM and Confocal Microscopies/Spectroscopies

**DOI:** 10.1038/s41598-020-75487-7

**Published:** 2020-10-22

**Authors:** Thales F. D. Fernandes, Oscar Saavedra-Villanueva, Emmanuel Margeat, Pierre-Emmanuel Milhiet, Luca Costa

**Affiliations:** Centre de Biochimie Structurale (CBS), CNRS, INSERM, Univ Montpellier, 34090 Montpellier, France

Correction to: *Scientific Reports* https://doi.org/10.1038/s41598-020-62529-3, published online 27 April 2020

The original version of this Article contained a typographical error in the spelling of the author Oscar Saavedra-Villanueva, which was incorrectly given as Oscar Saavedra V. This has now been corrected in the PDF and HTML versions of the Article, and in the accompanying Supplementary Information file.

