# Supplementary material for: Synchronous, Crosstalk-free Correlative AFM and Confocal Microscopies/Spectroscopies
Source: Sci Rep. 2020 Apr 27;10:7098. doi: 10.1038/s41598-020-62529-3 (PMC7184616; doi:10.1038/s41598-020-62529-3)
Supplement: Supplementary file 1 — Supplementary Information. [file 41598_2020_62529_MOESM1_ESM.pdf]

Synchronous, Crosstalk-free Correlative AFM and Confocal Microscopies/Spectroscopies - Supplementary Information

Thales F. D. Fernandes[1], Oscar Saavedra V.[1], Emmanuel Margeat[1], Pierre-Emmanuel Milhiet[1*], and Luca Costa[1**]

[1]Centre de Biochimie Structurale (CBS), CNRS, INSERM, Univ Montpellier, 34090, Montpellier, France.

[*]pem@cbs.cnrs.fr

[**]costa@cbs.cnrs.fr

March 18, 2020

1 Radiation Pressure

The radiation pressure acting upon the tip can be estimated by the change in momentum of the incident light. The radiation pressure P is defined as:

$$P = \frac{p}{c\pi R_L^2}, \tag{1}$$

where p is the laser power, c is the speed of light, and R_L is the size of the confocal spot. This pressure will act on the surface area of the AFM tip, πR_e^2, where R_e is the effective radius of the laser cross-section interaction with the tip apex and will give rise to a force. Typical values are $p \approx 500$ uW, $R_L =$ 250 nm, and $R_e =$ 1–2µm (estimated from Fig. 3 of the main manuscript), and thus resulting in a force of a few tens or hundreds of pN, which is in the

same order of magnitude as the ones measured in our experiments (Figs. 3a and 4b of the main manuscript).

2 Approach and retract curves of nanodiamond on a gold surface

In Fig. 6 from the main manuscript, the force curves are presented "unfolded", whereas the approach and retract curves are separated. Fig. 1d shows the conventional representation of a force curve with the approach and retract curves aligned as a function of nanodiamond-gold reciprocal distance: data were collected in a different region of the same gold substrate from Fig. 6 from the main manuscript. This helps to elucidate the fact that the system is behaving in a reversible way: approach and retract are highly correlated (superimposed) from either Figs. 1b and d. Fig. 1c shows a decay curve for the sum of all decays in Fig. 1a.

3 SLB lifetime

The presence of the tip (either ATEC or qp-BioAC) in the confocal spot results in a signal of low lifetime, < 1 ns (Fig. 4a from the main manuscript). Fig. 2a shows a decay curve from a DOPC region from Fig. 5c of the main manuscript, while Fig. 2b shows the respective phasor plot. Clearly, the decay curve presents no peak with low lifetime, indicating that the tip is not contributing with any spurious signal. This is further corroborated by the phasor plot showing data that belong to a single population and close to the semi-circle.

4 SEM image of qp-BioAC with plateau tips

As described in the Materials and Methods section of the main manuscript, the plateau tips are obtained from qp-BioAC cantilevers. An electron beam

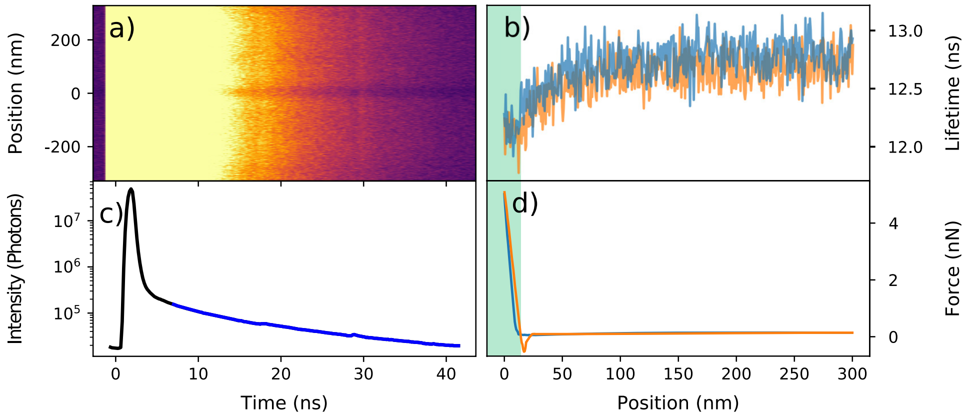

Figure 1: Simultaneous force curve and nanodiamond lifetime measurements approaching and retracting a gold substrate. (**a**) shows tip z-position versus time (decay curves). (**b**) shows the nanodiamond lifetime in function of tip position (approach and retract curves). (**c**) shows the decay curve for the sum of all decays in (**a**). (**d**) shows the force in function of tip position (approach and retract curves). In (**b** and **d**), cyan and orange represent approach and retract, respectively. A modified qp-BioAC with a nanodiamond on a 50 nm plateau at the tip's end was used with 90 µW laser power. The light blue region in (**b** and **d**) shows the contact regime between nanodiamond and gold.

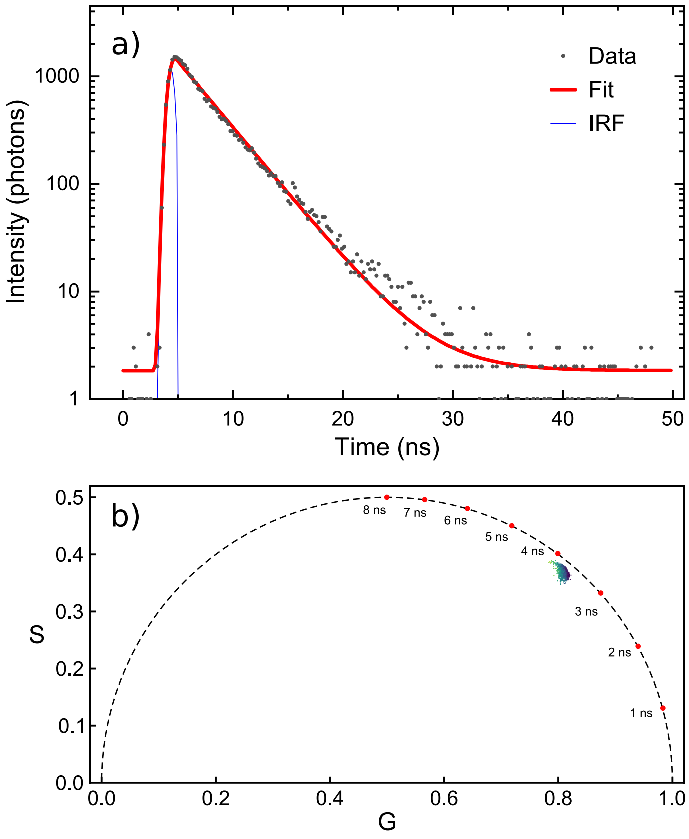

Figure 2: (**a**) Decay curve from a DOPC enriched region from Fig. 5c of the main manuscript: the blue curve is the Instrumental Response Function (IRF), black dots are experimental data and the red curve is the fit. (**b**) phasor plot of all DOPC/DPPC regions decays in Fig. 5c from the main manuscript. The power used was 120 nW, with an excitation filter of 488/10 nm and a band-pass emission filter of 525/39 nm. We used a qp-BioAC as AFM cantilever.

deposited carbon tip was grown on top of a thin layer of gold. Subsequently, a flat circular plateau of ≈ 50 nm diameter was obtained at the tip apex by means of FIB, as shown in a Scanning Electron Microscopy (SEM) image in Fig. 3.

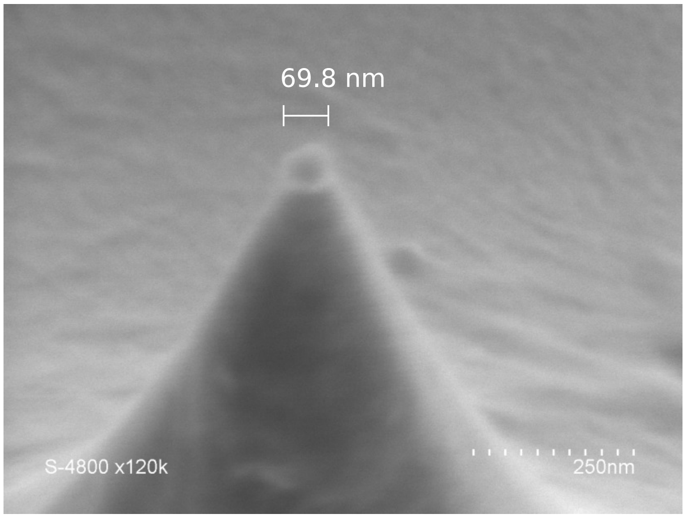

Figure 3: SEM image of a modified qp-BioAC plateau tip.

5 Lifetime distribution in DOPC/DPPC mixture

Fig. 5 of the main manuscript shows the DOPC/DPPC domains, labeled with BODIPY organic dyes, by AFM, fluorescence intensity and lifetime. Applying a mask on the AFM data, it is possible to separate the lifetimes from the two distinct enriched domains and get their distribution, which is shown in Fig. 4.

The BODIPY's lifetimes from DOPC and DPPC regions are 3.55 ± 0.07 ns and 3.71 ± 0.09 ns, respectively, leading to a ≈ 160 ps difference between the domains. This difference is in the same range as the one observed by Wu *et al.* for DOPC and DOPC-cholesterol mixtures in GUVs (ref. 38 in the main manuscript).

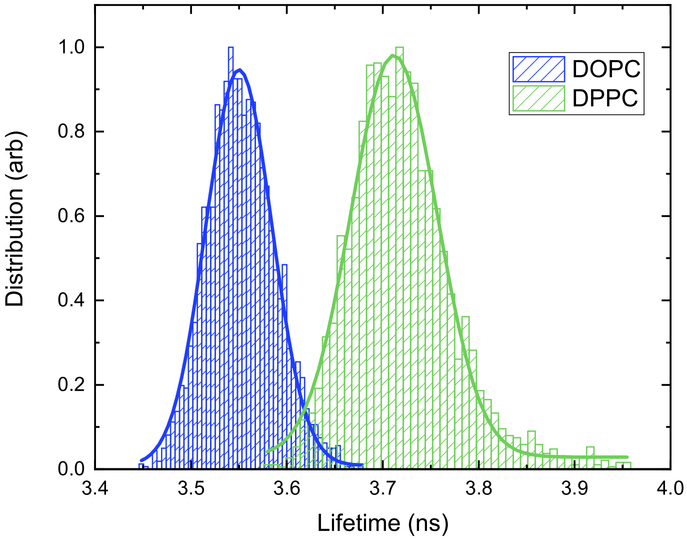

Figure 4: BODIPY lifetime distribution from DOPC (blue) and DPPC (green) domains from Fig. 5. The lifetimes of DOPC and DPPC are 3.55 ± 0.07 ns and 3.71 ± 0.09 ns, respectively.
